# Taurine Supplementation Alleviates Puromycin Aminonucleoside Damage by Modulating Endoplasmic Reticulum Stress and Mitochondrial-Related Apoptosis in Rat Kidney

**DOI:** 10.3390/nu10060689

**Published:** 2018-05-29

**Authors:** Alessandra Stacchiotti, Gaia Favero, Antonio Lavazza, Maria Monsalve, Luigi Fabrizio Rodella, Rita Rezzani

**Affiliations:** 1 Anatomy and Physiopathology Division, Department of Clinical and Experimental Sciences, University of Brescia, Viale Europa 11, 25123 Brescia, Italy; alessandra.stacchiotti@unibs.it (A.S.); gaia.favero@unibs.it (G.F.); luigi.rodella@unibs.it (L.F.R.); 2 Interdipartimental University Center of Research “Adaptation and Regeneration of Tissues and Organs (ARTO)”, University of Brescia, 25123 Brescia, Italy; 3 Istituto Zooprofilattico Sperimentale della Lombardia ed Emilia Romagna-IZSLER, 25124 Brescia, Italy; antonio.lavazza@izsler.it; 4 Instituto de Investigaciones Biomédicas “Alberto Sols” (CSIC-UAM), 28029 Madrid, Spain; mpmonsalve@iib.uam.es

**Keywords:** apoptosis, endoplasmic reticulum stress, kidney, puromycin aminonucleoside, taurine, ultrastructure

## Abstract

Taurine (TAU) is a sulfur-containing beta amino acid that is not involved in protein composition and anabolism, conditionally essential in mammals provided through diet. Growing evidence supports a protective role of TAU supply in osmoregulation, calcium flux, and reduction of inflammation and oxidant damage in renal diseases like diabetes. Endoplasmic reticulum (ER) stress, due to abnormal proteostasis, is a contributor to nephrotic syndrome and related renal damage. Here, we investigated the effect of dietary TAU (1.5% in drinking water for 15 days) in an established rat model that mimics human minimal change nephrosis, consisting of a single puromycin aminonucleoside (PAN) injection (intraperitoneally 15 mg/100 g body weight), with sacrifice after eight days. TAU limited proteinuria and podocytes foot processes effacement, and balanced slit diaphragm nephrin and glomerular claudin 1 expressions. In cortical proximal tubules, TAU improved lysosomal density, ER perimeter, restored proper ER-mitochondria tethering and mitochondrial cristae, and decreased inflammation. Remarkably, TAU downregulated glomerular ER stress markers (GRP78, GRP94), pro-apoptotic C/EBP homologous protein, activated caspase 3, tubular caspase1, and mitochondrial chaperone GRP75, but maintained anti-apoptotic HSP25. In conclusion, TAU, by targeting upstream ER stress separate from mitochondria dysfunctions at crucial renal sites, might be a promising dietary supplement in the treatment of the drug-resistant nephrotic syndrome.

## 1. Introduction

Nephrotic syndrome, which is one of the most widespread renal disorder in humans [1], includes a wide spectrum of diseases, with an idiopathic, toxic, or immunologic etiology, which is classified into four histological variants: minimal change disease (MCD), focal segmental glomerulosclerosis (FSGS), membranous nephropathy, and collapsing glomerulopathy, with different sensitivities to glucocorticoids [2]. MCD is the major idiopathic syndrome in children below one year of age, where corticosteroids, mainly prednisolone, are the choice treatment to ensure rapid remission [3]. Unfortunately, relapse occurs and a second line immunosuppressant with severe side effects may be necessary. In adults, MCD may progress into steroid-resistant FSGS [4,5,6]. However, an effective treatment to stop MCD recurrence and progression to end-stage damage is still debated in the scientific community along with its pathogenesis [7].

Puromycin aminonucleoside (PAN), which is an analogue of puromycin antibiotic, has been largely adopted to experimentally induce MCD in rodents (with a single high dose) [8] or FSGS (by repeated doses for over a longer period) [9]. Proteinuria is a common adverse event in primary podocytopathies due to the deranged filtration barrier that contributes to renal inefficiency [10]. Podocytes are terminally differentiated cells that are found in the visceral layer of the Bowman capsule, with pedicels separated by slit diaphragms, essential for pore formation [11]. PAN is a dose-dependent toxin for the podocytes induction of pedicels disorganization that is associated with in vivo and in vitro proteinuria [12].

Slit diaphragms in podocytes contain peculiar cytoskeletal proteins, like nephrin, podocin, and synaptopodin, regulated by actin and are essential for filtration [13]. In particular, nephrin, which is the main slit diaphragm glycoprotein, prevents the endoplasmic reticulum (ER) from being glycosylated, so ensuring permeability [14]. In contrast, abnormal nephrin traffic in slit diaphragms leads to heavy proteinuria [15]. Similar effects were linked to aberrant junctional claudin 1 in the glomeruli of transgenic mice and rats that were treated with PAN [16]. Moreover, in normal podocytes, the actin dynamic is modulated by calcium ions, but detrimental regulation of calcium based on calpain and calcineurin proteases occurred in PAN nephrosis [17,18].

Remarkably, calcium signaling and a proper balance between the secreted and misfolded proteins are key functions of the ER [19,20,21]. A strong relationship exists between proteinuria, renal damage and disrupted ER activity, called “ER stress”, and the unfolded protein response (UPR) [22].

Several papers outlined the main role of ER stress in the progression of acute and chronic kidney injury and diabetes in experimental models [23,24,25]. In MCD patients, ER stress was found to trigger tubule-interstitial fibrosis and inflammation linked to heavy proteinuria [26].

A key event in the UPR is the upregulation of resident ER chaperones glucose regulated protein 78 (GRP78, immunoglobulin heavy chain-binding protein, BiP), glucose-regulated protein 94 kDa (GRP94, also known as endoplasmin), and calreticulin and calbindin, which are all able to activate three molecular cascades to restore homeostasis [27,28]. However, if renal ER stress persists, apoptosis is triggered by mediators like C/EBP homologous protein (CHOP or GADD153) [29].

Heat shock proteins (HSPs) are highly conserved proteins that are involved in the maintenance of renal proteostasis in response to heat, hormones, drugs, heavy metals and ischemia [30,31]. HSPs include seven families, which are based on their common molecular weight, often essential for apoptosis regulation [32]. HSP25—in rodents (HSP27 in humans)—is a small HSPs member that protects actin cytoskeleton in podocytes foot processes and limits apoptosis during in vivo and in vitro PAN nephrosis [33,34]. GRP75 (also called mortalin or mthsp70), a member of HSP70 family, is predominant in mitochondria where it recovers respiratory enzymes during oxidative damage [35]. 

The kidney, which is only second to the heart in terms of mitochondria abundance, strongly adopts oxidative metabolism for hemodynamic and glomerular filtration [36,37]. In nephrotic syndrome and in the experimental PAN model, mitochondrial damage, reduced ATP production, and increased reactive oxygen species (ROS) have been reported [38,39]. 

Taurine (TAU) is a semi-essential beta amino acid present up to 0.1% in the human body, endogenously synthesized by cysteine, but is mainly introduced by a diet rich in sea-food and poultry [40]. TAU is necessary for the proper development of the infant brain, retina, and cardiac health [41] TAU transporter knockout mice exhibited overt oxidative damage, ER stress, and apoptosis [42]. A promising therapeutic role for TAU supplementation in metabolic diseases, like heart failure, diabetes, and muscle and inflammatory diseases, in rodent models but also in humans, has been recently reported by Schaffer and Kim [43]. TAU has also been rediscovered for the treatment of renal infections, nephrolithiasis and diabetes [44,45,46,47], but to the best of our knowledge, its role in acute PAN nephrosis is limited to a biochemical study by Venkatesan et al. [48].

Therefore, we completed this study to explore if ER stress in parallel to mitochondrial damage in podocytes and epithelial tubular cells can be potential targets for TAU in experimental PAN nephrosis in rats.

Our results outlined a novel therapeutic role for TAU dietary supplementation against ER stress and for mitochondria-driven events, like apoptosis and inflammation, in experimental PAN nephrotic syndrome coupled to massive proteinuria. 

## 2. Materials and Methods

### 2.1. Chemicals

Puromycin aminonucleoside was purchased by Sigma Aldrich (St. Louis, MO, USA). For monitoring urinary proteinuria, Multistix 10 SG Reagent Strips-Bayer was purchased by Siemens Healthcare Diagnostics (Milan, Italy). Histological stains for PAS, sodium metabisulphite, periodic acid and Harris or Mayer’s hematoxylin were of the highest grade (Sigma Aldrich, St. Louis, MO, USA). For immunohistochemistry, we purchased from Abcam (Cambridge, UK) monoclonal anti-CD68/ED1 (ab31630), polyclonal 4 hydroxy-2-nonenal (4HNE) (ab46545); polyclonal anti-caspase 3 activated (ab4051) and polyclonal anti-GRP78 (ab21685). From Sigma Aldrich (St. Louis, MO, USA), we purchased polyclonal anti-claudin 1 (SAB4200462), from GeneTex (Irvine, CA, USA) rabbit polyclonal anti-caspase 1-p10 (GTX123675) and from Santa Cruz Biotechnology, Inc. (Dallas, Texas, USA), we purchased rabbit polyclonal antibody anti-GRP75 (sc-13967), polyclonal anti-GADD153/CHOP (sc-575), goat polyclonal anti-HSP25 (sc-1049), goat polyclonal anti-nephrin (sc-32530), and rat monoclonal antibody anti-GRP94 (sc-56399).

### 2.2. Animal Treatment

Twenty two-month-old male Sprague Dawley (SD) rats were purchased from Harlan Laboratories Srl (San Pietro al Natisone, Udine, Italy). Animal care was performed according to the Italian Ministry of Health to comply with the commonly-accepted international guidelines known as “3Rs” European Community Council Directive 86/609/EEC.

SD rats were divided into the following four groups: (1) *n* = 4 SD rats fed standard rodent diet and tap water (control group); (2) *n* = 4 SD rats fed standard diet but drinking taurine (TAU) dissolved 1.5% in tap water for 15 days (TAU group) [49]; (3) *n* = 6 SD rats fed normal standard diet and injected with PAN (intraperitoneally injected; 15 mg/hg) (PAN group) [50] and (4) *n* = 6 SD rats fed standard diet injected with PAN, but drinking TAU one week before and another week after PAN injection (PAN plus TAU group). The rationale for two weeks of TAU supplementation was obtained from the study of Moloney et al. [51], where this duration of treatment reversed early diabetic damage in patients. The power analysis to define the number of animal for each group was evaluated by the Student *t* test with a significance of 0.05. All animals were sacrificed by cervical dislocation on day 8 after PAN injection, and the kidneys were removed for histopathology, immunohistochemical, and electron microscopy analysis. A similar approach was successful to best analyze stress response in the kidney in a previous study [52]. Proteinuria was measured at day 8 after PAN injection in 16 h collected urine samples during overnight placement in metabolic cages. Urinary parameters were assessed using Multistix 10SG Reagent Strips and the animals were considered to have a severe proteinuria following three consecutive readings >300 mg/dL [53].

### 2.3. Histopathology and Immunohistochemistry

For histopathology, the kidneys were fixed for immersion in 4% phosphate buffered paraformaldehyde for 48 h and were embedded in paraffin wax, as previously reported [54]. Paraffin sections (5 µm-thick) were prepared for PAS staining and immunohistochemistry. For quantitative analysis of glomerular tuft area, PAS stained renal sections were observed at a final magnification of 400× using a light microscope (Olympus, Hamburg, Germany) and at least 25 glomeruli for four different slides were randomly analyzed for each experimental group. The glomerular area was manually traced and estimated using imaging software (Image Pro Plus, Milan, Italy) by two investigators in a blinded manner. On the same section, also renal tubular-interstitial damage was also assessed on 10 non-overlapping fields at 200× and four different slides for each experimental group were randomly chosen. Normal cortical proximal tubules presented an epithelium with a single layer of cubic cells showing a purple PAS-positive brush border (consisting of microvilli during ultrastructural analysis) and a central nucleus, but these findings were lost in damaged tubules. Tubular atrophy, epithelial disruption, and protein casts were semi-quantitatively graded, as follows: grade 0, normal; grade 1, lesion area <25% of the field; grade 2, lesion area between 25% and 50% of the field; grade 3, lesion area between 51% and 75% of the field; and, grade 4, >75% lesion area. Tubular injury score represented the average of the grades that are assigned to the fields analyzed. The main changes examined include tubular atrophy, tubular casts, sloughing of tubular epithelial cells, and the presence of inflammatory cells, as previously reported by He et al. [55]. Serial paraffin sections were used for immunohistochemistry, dewaxed in xylene, rehydrated and subjected to antigen retrieval in 0.01 M citrate buffer (pH 6.0) in a microwave oven, and then treated in 3% hydrogen peroxide to block the endogenous peroxidase background. Slides were treated with normal serum of the same species producing the secondary antibody (diluted 1:5) for 1 h at room temperature in the dark and subsequently incubated, overnight at 4 °C in humidified chambers, with: rabbit polyclonal anti-GRP75 (diluted 1:200); rat monoclonal anti-GRP94 antibody (diluted 1:200); goat polyclonal anti-CHOP, anti-HSP25 and anti-nephrin antibodies (diluted 1:50-1:100-1:50 respectively), rabbit polyclonal anti-GRP78, anti-caspase3 and anti-caspase 1-p10 antibodies (diluted 1:250 and 1:50 respectively), mouse monoclonal anti-CD68/ED1 antibody (diluted 1:200); and, rabbit polyclonal 4HNE(diluted 1:400). Viewing was performed using the avidin-biotin-peroxidase complex (ABC-peroxidase kit, Vector Labs, Burlingame, CA, USA), according to manufacturer’s instructions. The peroxidase reaction was developed using 3′-3′-diminobenzidine tetrahydrochloride (Sigma Aldrich, St. Louis, MO, USA) as the substrate and hydrogen peroxide as the catalyst for 7 min in the dark. To assess the specificity of each immunoreaction, we omitted primary antibodies and substituted those with tris buffered solution. All sections were counterstained by hematoxylin, dehydrated and mounted for light microscopy analysis (Olympus, Hamburg, Germany). Then, an immunopositive area was quantified at 400× using computerized image analysis software (Image Pro Plus, Milan, Italy), as follows: brown peroxidase-positive area for each field was manually circled, then automatically converted into a gray-scale pixel to obtain a total value as arbitrary value (AU). Twenty randomly chosen non-overlapping fields for each immunostaining were estimated. In particular, for the quantification of nuclear markers, nuclei that were further stained with haematoxylin appeared blue-violet and were not estimated by the software set on brown color.

### 2.4. Transmission Electron Microscopy

The second kidney was fragmented in small pieces and was fixed for immersion in 2.5% glutaraldehyde in cacodylate buffer 0.1 M for 3 h then post-fixed in 2% osmium tetroxide in the same buffer for 1 h at 4 °C. Dehydration in progressive ethanol concentrations, propylene oxide, and embedding in Epon 812 mixture were performed, as previously described [56].

Semithin sections (1 µm thick) obtained with an UltraCut E ultra-microtome were then stained by methylene blue-azure II, and then observed under a light microscope to verify the presence of lysosomes at 100×. Subsequently, thin sections (80 nm thick) collected on cupper grids were observed under a transmission electron microscope Tecnai G2 Spirit (FEI Company, Eindhoven, The Netherland) at 80 kV. Morphometric computerized analysis of foot processes of podocytes was performed at 15,000×, but the ER perimeter was estimated at 26,000×, and ER-mitochondria distance at 135,000× by two observers blinded of the treatment, as previously described [57].

### 2.5. Statistical Analysis

All data are expressed as mean ± standard deviation and analysis of the statistical significance was obtained using one-way analysis of variance (ANOVA) corrected by Bonferroni test to compare the variability of a group with all the other experimental groups and *p* < 0.05 being considered to be significant. All of the experiments were performed in triplicate, and data were collected and analyzed by Origin Pro 9.1 software (Origin Lab Corporation, Northampton, MA, USA).

## 3. Results

### 3.1. Metabolical and Histopathological Effects of TAU in Nephrotic Rats

Similar metabolic and histological data were recorded in the control and TAU only treated rats, so they are labelled the “control group” in the next described evaluation. Metabolic and urinary parameters are summarized in Table 1. PAN-treated rats developed massive proteinuria (1200 ± 130 mg/dL) compared with the control group in which proteinuria was undetectable. However, in the TAU supplemented PAN group, proteinuria significantly decreased to a low range (80 ± 22 mg/dL). Urinary volume tripled in PAN nephritic rats, but significantly decreased in the TAU supplemented PAN group. Urinary leukocytes were absent in the control group, abundant in PAN group (125/dL), and decreased after PAN plus TAU treatment (70/dL) (all *p* < 0.05). To further corroborate the role of TAU preconditioning in the PAN model, the renal expression of the lipid peroxidation marker 4HNE was analyzed and quantified (Figure S1).

**Figure 1:**
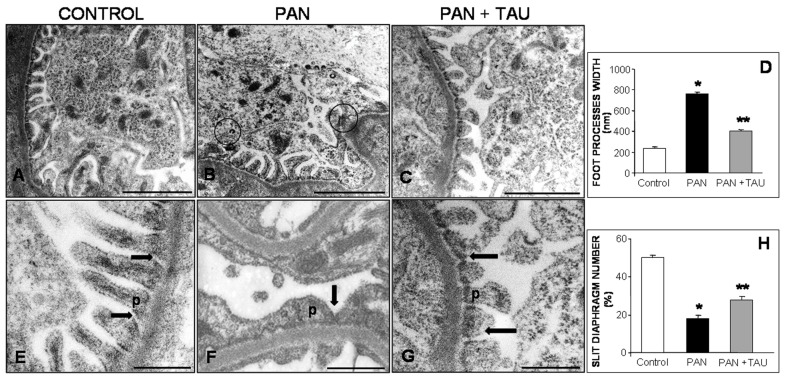
Podocyte damage. Dietary TAU reduced podocyte damage in PAN. Transmission electron micrographs of glomerular podocytes in (**A**–**E**) control, (**B**–**F**) PAN-injected rats, or (**C**–**G**) with TAU supplementation. In the PAN group, podocytes showed tight junctions (circled) together with pedicels feet (p) fusion and reduced slit diaphragm number (arrow). These changes were improved by TAU pre-treatment and were almost similar to control rats. (**A**–**C**) bar represents 1 µm and (**D**–**F**) bar represents 500 nm. Quantitative analysis of (**D**) foot processes width and (**H**) slit diaphragm number. Data are expressed as means or percentages. * *p* < 0.05 significant versus control group; ** *p* < 0.05 significant versus PAN group.

PAS staining did not reveal any damage to glomeruli, and similarly no differences in glomerular area were observed in experimental groups (data not shown). These findings confirmed the hallmark of “minimal change nephrosis” (MCD), i.e., absence of glomerular damage under light microscopy analysis. So, we analyzed the filtration barrier by transmission electron microscopy, the elective method that best defines proteinuria-induced changes in MCD patients [58]. In control rats, regular podocytes’ foot processes adhered to the basal membrane and well-structured slight diaphragms were visualized (Figure 1A,E). In contrast, in PAN-injected rats, prominent fusion of pedicels and tight junctions between pedicels, together with limited slight diaphragms, were observed (Figure 1B,F). In contrast, after TAU pre-treatment, more regular thin foot processes and recovered filtration slits in the glomerular barrier were evident (Figure 1C,G). Estimation of foot processes width on electron micrographs demonstrated a significant enlargement in PAN-injected rats (750 nm) when compared to the control group (200 nm), but a decreased width in the PAN supplemented with TAU group (Figure 1D). These data indicated that TAU intake partially improved podocytes effacement in the PAN-treated group. Slight diaphragms quantification significantly decreased by 42% in the PAN group (*p* < 0.05), but increased by 21% in PAN plus TAU supplemented rats (Figure 1H). These values indicated that TAU pre-treatment positively influenced the distribution of glomerular filtration sites.

### 3.2. TAU Changes Podocytes Markers Distributions in Nephrotic Rats

**Figure 2:**
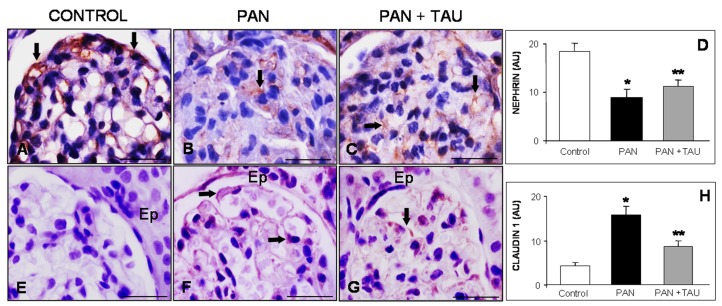
Glomerular cytoskeletal markers. (**A**–**C**) Nephrin and (**E**–**G**) claudin 1 in PAN nephrotic rats are reversed by TAU taurine pre-treatment. Arrows indicate strong nephrin immunostaining in (**A**) control, (**B**) scattered in PAN group, and (**C**) condensed after TAU supply. Claudin 1 staining was (**E**) absent in control rats, (**F**) evident (arrows) in glomeruli and epithelial parietal (Ep) cells in PAN group, and (**G**) weak in TAU pre-treated rats. Bar represents 20 µm. Quantitative evaluation of (**D**) nephrin and (**H**) claudin 1 immunostainings in glomeruli are expressed as arbitrary units. * *p* < 0.05 statistically significant versus control group; ** *p* < 0.05 significant versus PAN group.

To confirm podocyte impairment using transmission electron microscopy, we explored the glomerular localization of the cytoskeletal markers nephrin and claudin 1 to highlight the extent of PAN nephrosis in different groups. Nephrin, which is a structural marker of podocyte foot processes and the main component of a normal slight diaphragm, was highly expressed in control rats (Figure 2A), diminished in PAN nephrotic rats (Figure 2B), was partially restored in the PAN group receiving TAU supplementation (Figure 2C). Estimation of nephrin staining intensity, as arbitrary units, was high in the control group, lower in the PAN group (*p* < 0.05), and then increased in the PAN group receiving TAU (Figure 2D).

In contrast, claudin 1, which is a tight junctional marker that is localized in the external epithelial layer of Bowman capsule, was barely detectable in the control group (Figure 2E), markedly expressed in PAN group (Figure 2F), and significantly reduced at the glomerular level in the PAN supplemented TAU group (Figure 2G). The immunoreaction was also quantified and the relative immunopositivity evaluation is summarized in Figure 2H.

### 3.3. TAU Reduces Glomerular ER Chaperones and Apoptosis

**Figure 3:**
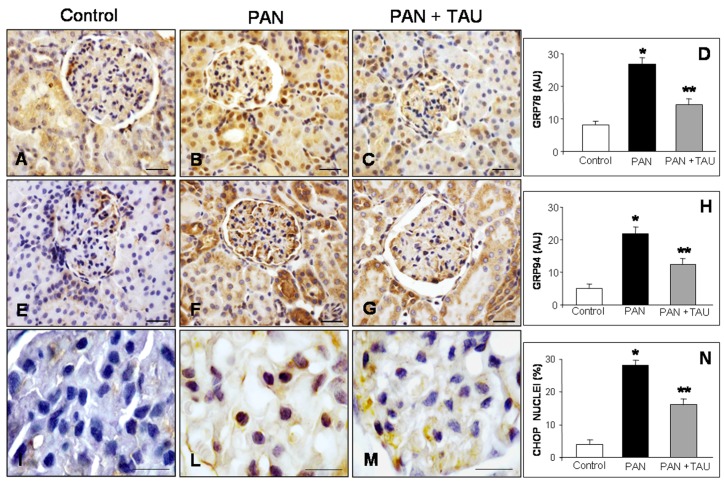
Endoplasmic reticulum stress makers. (**A**–**C**) GRP78, (**E**–**G**) GRP94, (**I**–**M**) C/EBP homologous protein (CHOP) in (**A**,**E**,**I**) control rats, (**B**,**F**,**L**) PAN nephrotic rat model are ameliorated by (**C**,**G**,**M**) TAU pre-treatment. Bar represents 20 µm. Quantitative analysis of immunostaining for (**D**) GRP78 and (**H**) GRP94, expressed as arbitrary units, and (**N**) of nuclear positive percentage of CHOP immunostaining. * *p* < 0.05 statistically significant versus control group; ** *p* < 0.05 significant versus PAN group.

ER stress response was assessed by the immunohistochemistry analysis of the glomerular expression of ER resident chaperones in all of the experimental groups. With this approach, the master ER chaperone GRP78, was weakly or moderately detected in glomeruli and surrounding cortical distal tubules in control rats (Figure 3A). In contrast, in PAN-treated nephrotic rats, a marked signal was detected in glomeruli and proximal tubules also at the nuclear level (Figure 3B), but decreased in the proximal tubules in TAU pre-treatment (Figure 3C). Estimation of the GRP78 signal doubled in the PAN nephrotic group and significantly decreased by 60% in PAN-injected rats supplemented with TAU (Figure 3D). To confirm the evidence of ER stress, GRP94 localization was further assessed. GRP94 immunostaining, barely detectable in control rats (Figure 3E), was strongly expressed in glomeruli and proximal tubules of PAN-treated rats (Figure 3F), but decreased in the PAN group that was supplemented with TAU (Figure 3G). Quantification of GRP94 immunoreaction showed a decrease in PAN nephrotic rats that were supplemented with TAU (Figure 3H). Furthermore, to better examine the influence of ER stress on apoptosis, we examined the glomerular expression of CHOP. Remarkably, CHOP was undetectable in the control group (Figure 3I), evident in podocyte nuclei in the PAN-injected rats (Figure 3L), and decreased after TAU supplementation (Figure 3M). Quantitative analysis of CHOP positive nuclei, expressed as percentage, was 4% in the control group, about 28% in the PAN nephrotic rats, and 16% in the PAN group that was treated with TAU (Figure 3N). These data clearly demonstrated that TAU limited glomerular ER stress and the pro-apoptotic cascade.

### 3.4. TAU Decreases Tubular Apoptosis and Oxidative Damage

**Figure 4:**
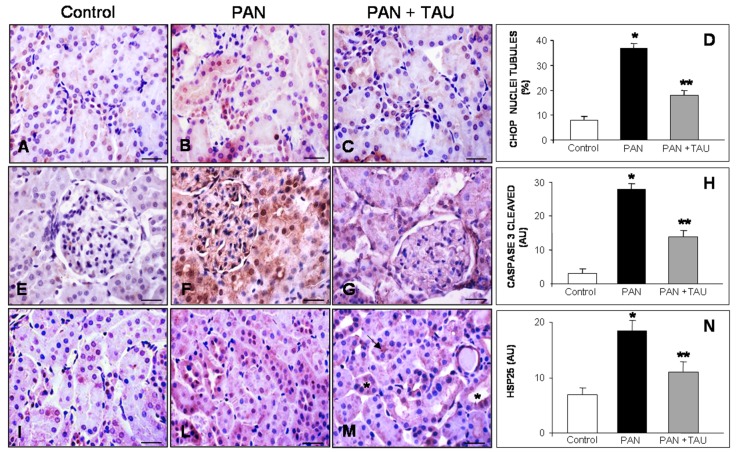
Proximal tubular apoptosis. (**A**–**C**) CHOP, (**E**–**G**) activated caspase 3, (**I**–**M**) HSP25 in (**A**,**E**,**I**) control rats, and (**B**,**F**,**L**) PAN nephrotic rats that decreased with oral TAU pre-treatment (**C**,**G**,**M**). (**M**): the arrow shows the brush border positivity at proximal tubule level and the asterisks identify the positivity in the distal tubules. Bar represents 20 µm. Quantitative analysis of immunostaining for (**D**) CHOP, (**H**) expressed as nuclear positive percentage, and (**N**) for caspase 3 cleaved and HSP25, expressed as arbitrary units. * *p* < 0.05 statistically significant versus control group; ** *p* < 0.05 significant versus PAN group.

While considering the crucial role of the first tract of the nephron in the reabsorption of excessive urinary proteins, we extended immunohistochemical analysis of ER-driven apoptosis to proximal cortical tubules. Occasional pro-apoptotic CHOP in the nuclei of the proximal tubules of control rats (Figure 4A) was clearly expressed in disrupted proximal tubules in PAN-injected rats on day eight (Figure 4B) and decreased in the PAN plus TAU group (Figure 4C). Accordingly, the percentage of nuclear positivity shifted from 8% in control rats to 36% in the PAN group and to 18% in TAU supplemented group (Figure 4D). 

Caspase 3 is the crucial marker of the final step in the apoptotic cascade that must be activated by proteolysis [59]. So, we evaluated the presence of cleaved caspase 3 in cortical renal tubules in our model. Control rats did not express cleaved caspase 3 in the kidney (Figure 4E); in contrast, in the PAN group, an intense nuclear staining was observed in the proximal tubules, but only occasionally in the glomeruli on day eight (Figure 4F). After TAU pre-treatment, cleaved caspase 3 immunostaining was limited to a few nuclei in the proximal tubules (Figure 4G). Quantitative analysis showed that nuclear positivity for the cleaved activated caspase 3 was very low in control rats, 26 AU in the PAN group, but decreased in rats that were receiving PAN and TAU (*p* < 0.05) (Figure 4H).

To best correlate the extent of apoptosis to oxidative damage, we visualized the renal distribution of HSP25, an inducible actin chaperone able to oppose ATP-depletion in the kidney. Nuclear HSP25 expression was weak in the proximal tubules of the control rats (Figure 4I), and intense in the PAN-treated group localized in the proximal and distal tubules (Figure 4L). In contrast, in PAN nephrotic rats supplemented with TAU, the HSP25 signal shifted to the brush border in proximal tubules and to the cytoplasm of the distal tubules (Figure 4M). Quantitative analysis of HSP25 abundance confirmed the highest value in PAN-injected rats, which decreased by 50% after TAU supplementation to a value that was not that different from the control (*p* < 0.05) (Figure 4N). These results indicated that dietary TAU supplementation reduced tubular apoptosis and oxidative damage in proximal tubules.

### 3.5. TAU Improves Tubular Inflammation and Mitochondrial Stress in PAN Nephrosis

In addition to apoptosis and ER stress, tubular inflammation and the interstitial recruitment of macrophages from circulation are also involved in the renal lesions that are induced by acute proteinuria.

**Figure 5:**
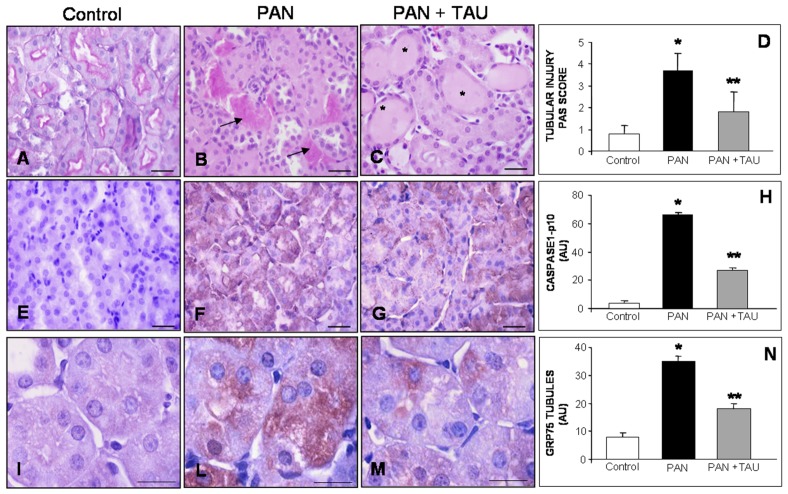
Tubular Inflammation and Mitochondrial Stress. (**A**–**C**) PAS histopathology, (**E**–**G**) activated caspase 1-p10 and (**I**–**M**) mitochondrial chaperone GRP75 stainings in the proximal tubules of (**A**,**E**,**I**) control rats, (**B**,**F**,**L**) PAN nephrotic rats and (**C**,**G**,**M**) PAN rats orally pre-treated with TAU. (**A**–**C**) The arrows identify the proteinaceous casts and the asterisks show the tubular dilatation. Bar represents 20 µm. Tubular damage expressed as (**D**) tubular injury score, (**H**) quantitative estimation of caspase 1-p10, (**N**) and GRP75 immunostainings, expressed as arbitrary units. * *p* < 0.05 statistically significant versus control group; ** *p* < 0.05 significant versus PAN group.

In our nephritic model, the histological analysis of PAS stained cortical tubules revealed a regular purple brush border in the control rats (Figure 5A), PAS stained proteinaceous casts in the lumen of the PAN nephrotic group (Figure 5B), and enlarged dilated proximal tubules in the PAN group receiving TAU (Figure 5C). Semi-quantitative estimation of proximal tubular damage, expressed as a “tubular score”, was 0.5 in control rats, significantly increased to 3.5 in nephrotic rats, and decreased to 2 AU in PAN group pre-treated with TAU (Figure 5D).

Moreover, we decided to examine the tubular presence of activated caspase 1, a component of NLRP3 inflammasome, which is the multiprotein complex sensitive to ER damage. The staining of activated caspase 1, i.e., the subunit caspase 1-p10, was barely detectable in the proximal tubules in control rats (Figure 5E), intense in the proximal tubules in the PAN-injected group (Figure 5F), and weaker in the PAN rats that received TAU (Figure 5G). Quantitative analysis revealed that caspase1 p10 intensity was very low in the control group, dramatically increased 14 fold in PAN, but significantly decreased in the PAN plus TAU group, *p* < 0.05 (Figure 5H). Figure S1 shows the evaluation of the CD68/ED1 macrophage infiltration in the tubule interstitial area.

Finally, to better understand the effect of different treatments on mitochondrial homeostasis, we analyzed the expression of the mitochondrial chaperone GRP75. In control rats, GRP75 immunoreaction was faint in the cytoplasm of the proximal tubules (Figure 5I), strong in the damaged proximal tubules of the PAN nephrotic rats (Figure 5L), and limited to few epithelial cells in the PAN rats supplemented with TAU (Figure 5M). Estimation of GRP75 immunostaining tripled in the PAN group as compared with the control group, but halved in PAN rats that were supplemented with TAU (value 18 AU; *p* < 0.05) (Figure 5N). Therefore, all of these data indicated that TAU pre-treatment was effective against “sterile” inflammation and oxidative damage by modulating caspase 1 activation and GRP75 abundance in proximal tubules.

### 3.6. Effect of TAU on Mitochondria and Rough ER in Nephrotic Proximal Tubules

**Figure 6:**
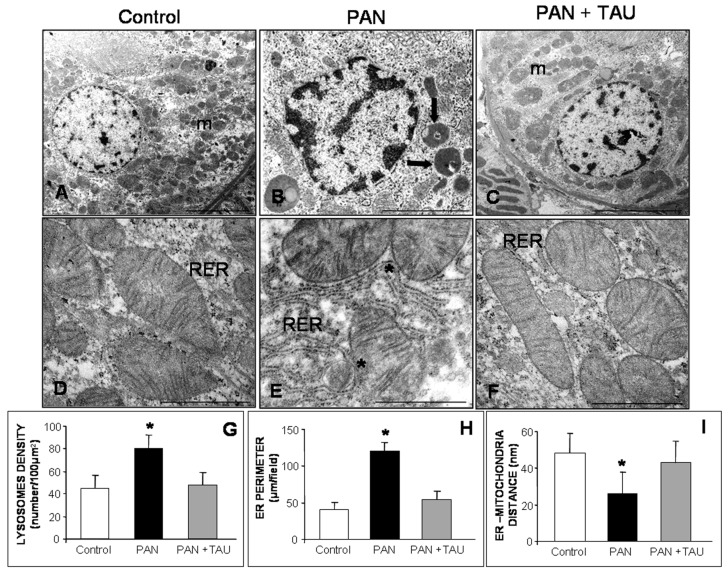
Ultrastructural evaluation at cortical proximal tubular level**.** (**A**,**D**) Lysosomes and mitochondria in cortical proximal tubules-S1 segment in control, (**B**,**E**) PAN nephrotic rats and (**C**,**F**) PAN rats pre-treated with TAU. (**m**): mitochondria; (RER): rough endoplasmic reticulum; arrow identifies lysosomes and asterisks identify the contact between organelles. (**A**–**C**) bars represent 1 µm and (**D**–**F**) bars represent 500 nm. Quantitative estimation of (**G**) lysosomal density, (**H**) RER perimeter, (**I**) lysosomes-mitochondria juxtaposition. * *p* < 0.05 versus control and PAN pre-treated with TAU groups.

To understand whether TAU alleviation of tubular apoptosis and altered oxidative status that we observed via immunohistochemistry may be correlated to the morphology of the ER, mitochondria, or other organelles, we examined the proximal tubules using electron microscopy. At low magnification, we observed scattered dense mitochondria and scarce lysosomes in the control group (Figure 6A), excessive perinuclear lysosomes, and multiple rough ER (RER) cisternae in the PAN group (Figure 6B), and elongated mitochondria in the PAN plus TAU supplemented group (Figure 6C). At high magnification, we focused on mitochondria and RER features. In the control proximal tubules, mitochondria displayed parallel cristae and a regular distance from RER (Figure 6D). In contrast, in the PAN nephrotic group, the mitochondria contained a few short cristae, a disrupted outer membrane, and were fused in multiple points with RER (Figure 6E). Remarkably, in the PAN rats that were supplemented with TAU, mitochondria presented restored cristae, well-defined outer membrane, and regular RER distance that was similar to the control rats (Figure 6F).

To best corroborate the above findings, lysosomal density, RER perimeter, and the distance between ER and the outer mitochondrial membrane were measured. In proximal tubules of control rats, lysosomal density was 45 (expressed as number per 100 µm^2^), almost doubled (80) in PAN injected rats, but decreased to the control level after TAU supplementation (48) (Figure 6G). In PAN-treated rats, ER perimeter significantly increased up to 120 µm/field in proximal tubules when compared to the control group (40 µm/field). After TAU pre-treatment, the ER perimeter was similar to that of the control rats (44 µm/field) (Figure 6H). Furthermore, when considering the crucial role of ER and mitochondria juxtaposition in regulating ER signaling [60], we estimated ER-mitochondria tethering in our experimental model. The distance between organelles in control rats was less than 50 nm, significantly decreased to 26 nm in the PAN-injected group, but was almost similar to the control value in TAU supplemented PAN rats (43 nm) (all *p* < 0.05) (Figure 6I). These results indicated that TAU inhibited mitochondrial alterations and it contributed to the maintenance of a proper distance between mitochondria and RER in proximal tubules. 

## 4. Discussion

Proteinuria, which is the presence of proteins in urine, is not only a diagnostic marker of glomerular damage but also a strong contributor to tubular decline and the progression of chronic kidney disease [61,62]. ER stress and UPR are fundamental mechanisms that are involved in the pathogenesis of proteinuric renal failure [25,63]. However, ER modulation by drugs may be of great value for a nephrologist to develop effective personalized ER-targeted treatments in unresponsive or recurrent nephrotic syndrome, instead of conventional glucocorticoids or immunosuppressive drugs [7,64].

One major point addressed in this study was that TAU alleviated experimental PAN nephrosis and proteinuria in rats by reducing ER stress and HSPs response.

Indeed, in rats, a single-dose PAN injection induced massive proteinuria on day eight, which was associated with ultrastructural glomerular and tubular alterations, mimicking human MCD [48]. In rats, TAU preconditioning, one week before and another after PAN injection, decreased proteinuria and renal damage. Through ultrastructural analysis, we clearly observed reduced podocyte effacement in the TAU supplemented nephrotic animals, but an enhanced slit diaphragm number and distribution. Furthermore, in agreement with morphological findings, TAU restored glomerular nephrin, which is the main slit diaphragm component, and minimized claudin 1 in our nephrotic model.

Notably, claudin 1 in nephrotic glomeruli was correlated with the presence of tight junctions, and it indicated a dysfunctional filtration barrier able to promote proteinuria in line with the findings that were reported by Gong et al. [16]. However, in this study, we demonstrated that TAU dietary supplementation restored proper expressions of these two markers in the PAN nephrotic model.

In vitro studies on malignant cells reported that PAN induced ER stress by translocon opening in the ER membrane, attracting proteins in the lumen and releasing calcium ions in the cytoplasm [65]. Moreover, also in PAN-treated podocytes, abnormal calcium flux, calpain, and calcineurin were described [18]. GRP78 and GRP94 expression was detected in our study in PAN nephrotic rats, which agrees with the findings that were reported by Inagi et al. [19]. However, TAU preconditioning inhibited ER stress in nephrotic glomeruli and proximal tubules in line with previous studies on cisplatin or starvation-triggered ER damage [66,67].

Another interesting novel finding of this study is the attenuation of ER stress driven by pro-apoptotic CHOP and HSP25 in glomeruli and proximal tubules in PAN nephrotic rats pre-treated with TAU. Although apoptosis and oxidative damage that was linked to ER stress in kidneys treated with PAN have been well established [68,69], the beneficial role of TAU in nephritic kidney is still unclear. However, TAU hampered ER and mitochondrial damage in neurodegeneration and stroke [70].

As podocytes are the main target of PAN toxin, proximal tubular cells may also be involved in the reabsorption of excessive urinary proteins from the ultrafiltrate by unique transporters in their apical site, called the brush border [38]. However, lysosomal cathepsins are also directly involved in acute kidney disease progression [71]. Therefore, we analyzed cortical proximal tubules in PAN nephrotic model while focusing on ER stress, apoptosis, and subcellular organelles changes in the lysosomes and mitochondria. Intriguingly, our findings on the maintenance of cortical tubular dilatations in PAN pre-treated with TAU mice agreed with those of Mozaffari et al. [72] who reported an apparent detrimental role of TAU in rat kidney after ischemia-reperfusion.

Notably, we demonstrated that TAU antagonized tubular ER stress and apoptosis in nephrotic animals. Moreover, TAU improved abnormal lysosomal accumulation, ER mitochondria tethering, and mitochondrial integrity reducing HSP25 and GRP75 expressions. These findings highlighted the preventive effect of TAU in subcellular sites essential for oxidative metabolism and calcium flux, and that HSPs may be therapeutic targets [73,74]. In addition, the recovery of proper ER-mitochondria tethering in TAU supplemented nephrotic rats suggested that TAU modulated bioenergetics [75] or worked as a mitochondrial buffer of calcium in the nervous system, according to El Idrissi [76]. Furthermore, in MELAS (mitochondrial myopathy, encephalopathy, lactic acidosis, and stroke-like episodes), which is a human mitochondrial disease with stroke-like episodes, TAU supplementation recovered mitochondrial respiratory function revealing a promising therapeutic role [43]. Finally, we showed that oral TAU reduced tubular inflammation and caspase 1 activation in PAN nephrosis. Recent studies reported that NOD-like receptor protein 3 (NLRP3) inflammasome was overloaded with calreticulin in the ER in proximal tubular cells that were challenged with albumin [77]. ER stress triggered a sterile inflammatory cascade, activating NLRP3 and caspase 1 in proteinuric patients [78]. Consequently, we hypothesized that dietary TAU supplementation, improving ER homeostasis, may be able to limit the inflammatory reaction in proximal renal tubules, even if further experiments are needed to verify this hypothesis.

## 5. Conclusions

**Figure 7:**
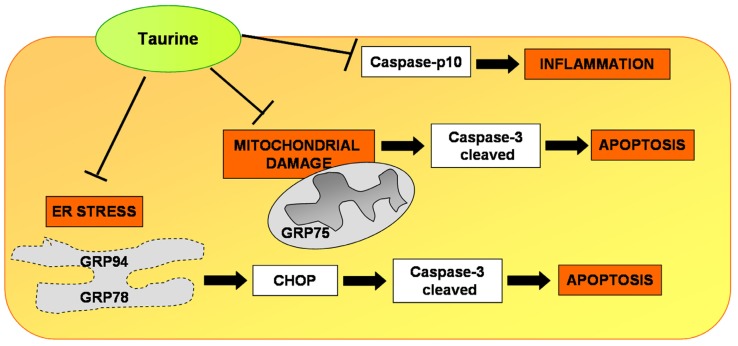
Potential protective effects of dietary taurine in puromycin aminonucleoside nephrosis in rats**.** Endoplasmic reticulum stress and mitochondrial-related apoptosis were limited by taurine inhibition of CHOP and activated cleaved caspase 3. T-bars indicate inhibition, and arrows indicate activation. (ER): endoplasmic reticulum; (CHOP): C/EBP homologous protein.

Taken together, our results suggest that dietary TAU significantly preserves ER and mitochondria features, promoting glomerular and tubular recovery and reducing experimental puromycin-induced damage in rat kidney. Intriguingly, TAU decreased ER stress, and in parallel, mitochondria-related apoptosis, drives mitochondria and ER expression of specific stress markers, hampering puromycin nephrotic damage (Figure 7). This study reveals that CHOP may be an upstream therapeutic TAU target able to drive downstream apoptosis triggered by ER and mitochondrial UPR in the puromycin model [79]. Finally, this translational study implies that TAU might act as a potential promising dietary additive in drug resistant-MCD patients. We only described the effective role of TAU in rat; new studies on this potentiality should be completed in the future.

## Figures and Tables

**Table 1:** Metabolic and urinary data of puromycin (PAN) nephrotic rats with either taurine (TAU) or no TAU treatment.

	Control (*N* = 4)	Taurine (*N* = 4)	PAN (*N* = 6)	PAN + Taurine (*N* = 6)
Body weight (g)	248.5 ± 10.0	240.8 ± 10.0	200.6 ± 12.00 *	230.5 ± 10.0 **
Urinary Volume (mL/day)	5.5 ± 0.9	5.3 ± 0.9	18.8 ± 10.0 *	9.5 ± 8.5 **
Proteinuria	ND	ND	3+	1+
Urobilinogen (mg/dL)	0.2	0.2	0.2	0.2
Hemoglobin (mg/dL)	0.025	0.025	0.025	0.025
Leukocytes (N/dL)	absent	absent	125 (2+)	70 (2+)

Note: Values are means ± SD, * *p* < 0.05 versus control and taurine groups, ** *p* < 0.05 versus PAN group; 24 h urine was analyzed by MULTISTIX 10SG reagent strips (Bayer). Proteinuria: ND, less than 15 mg/dL, (1+), 30–100 mg/dL; (2+), 100–300 mg/dL, (3+), 300–2000 mg/dL, Glucose: ND less than 75 mg/dL; Ketones: ND less than 5 mg/dL in all the groups; Urobilinogen: Normal value 0.2.

## Supplementary Materials

The following are available online at http://www.mdpi.com/2072-6643/10/6/689/s1, Figure S1: 4HNE and CD68/ED1 renal expression.

## References

[1] Pal A., Kastel F. (2016). History of nephrotic syndrome and evolution of its treatment. Front. Pediatr..

[2] Barisoni L., Schnaper H., Kopp J. (2007). A proposed taxonomy for the podocytopathies: A reassessment of the primary nephritic diseases. Clin. J. Am. Soc. Nephrol..

[3] Vivarelli M., Massella L., Ruggiero B., Emma F. (2017). Minimal Change Disease. Clin. J. Am. Soc. Nephrol..

[4] Teeninga N., Kist van Holthe K., Nauta J. (2012). Extending prednisolone treatment does not reduce relapses in childhood nephrotic syndrome. J. Am. Soc. Nephrol..

[5] Hogan J., Radhakrishnan J. (2013). The treatment of minimal change disease in adults. J. Am. Soc. Nephrol..

[6] Mathieson P. (2007). Minimal change nephropathy and focal segmental glomerulosclerosis. Semin. Immunopathol..

[7] Greka A. (2016). Human genetics of nephrotic syndrome and the quest for precision medicine. Curr. Opin. Nephrol. Hypertens..

[8] Shen X., Jiang H., Ying M., Xie Z., Li X., Wang H., Zhao J., Lin C., Wang Y., Feng S. (2016). Calcineurin inhibitors cyclosporine A and tacrolimus protect against podocyte injury induced by puromycin aminonucleoside in rodent models. Sci. Rep..

[9] Pippin J., Brinkkoetter P., Cormack-Aboud F., Durvasula R., Hauser P., Kowalewska J., Krofft R., Logar C., Marshall C., Ohse T. (2009). Inducible rodent models of acquired podocyte diseases. Am. J. Physiol. Ren. Physiol..

[10] Haraldsson B., Nystrom J., Deen W. (2008). Properties of the glomerular barrier and mechanisms of proteinuria. Physiol. Rev..

[11] Kriz K., Shirato I., Nagata M., LeHir M., Lemiev K. (2013). The podocyte’s response to stress: The enigma of foot process effacement. Am. J. Physiol. Ren. Physiol..

[12] Burford J., Gyarmati G., Shirato I., Kriz W., Lemiev K., Peti-Peterdi J. (2017). Combined use of electron microscopy and intravital imaging captures morphological and functional features of podocyte detachment. Pflugers Arch. Eur. J. Physiol..

[13] Welsh G., Saleem S. (2011). The podocyte cytoskeleton-key to a functioning glomerulus in health and disease. Nat. Rev. Nephrol..

[14] Yan K., Khoshnoodi J., Ruotsalainen V., Tryggvason K. (2002). N-linked glycosylation is critical for the plasma membrane localization of nephrin. J. Am. Soc. Nephrol..

[15] Swiatecka-Urban A. (2017). Endocytic trafficking at the mature podocyte slit diaphragm. Front. Pediatr..

[16] Gong Y., Sunq A., Roth R., Hou J. (2017). Inducible expression of claudin-1 in glomerular podocytes generates aberrant tight junctions and proteinuria through slit diaphragm destabilization. J. Am. Soc. Nephrol..

[17] Greka A., Mundel P. (2012). Regulation of podocyte actin dynamics by calcium. Semin. Nephrol..

[18] Ding F., Li X., Li B., Guo J., Zhang Y., Ding J. (2016). Calpain-mediated cleavage of calcineurin in puromycin aminonucleoside-induced podocyte injury. PLoS ONE.

[19] Inagi R., Ishimoto Y., Nangaku M. (2014). Proteostasis in endoplasmic reticulum-new mechanisms in kidney disease. Nat. Rev. Nephrol..

[20] Guerriero C., Brodsky J. (2012). The delicate balance between secreted protein folding and endoplasmic reticulum-associated degradation in human physiology. Physiol. Rev..

[21] Krebs J., Agellon L., Michalak M. (2015). Ca^2+^ homeostasis and endoplasmic reticulum (ER) stress: An integrated view of calcium signaling. Biochem. Biophys. Res. Commun..

[22] Taniguchi M., Yoshida H. (2015). Endoplasmic reticulum stress in kidney function and disease. Curr. Opin. Nephrol. Hypertens..

[23] Inagi R. (2010). Endoplasmic reticulum stress as a progression factor for kidney injury. Curr. Opin. Pharmacol..

[24] Cybulsky A. (2017). Endoplasmic reticulum stress, the unfolded protein response and autophagy in kidney diseases. Nat. Rev. Nephrol..

[25] El Karoui K., Vian A., Dellis O., Bagattin A., Nguyen C., Baron W., Burtin M., Broueilh M., Heidet L., Mollet G. (2016). Endoplasmic reticulum stress drives proteinuria-induced kidney lesions via lipocalin 2. Nat. Commun..

[26] Lindenmeyer M., Rastaldi M., Ikehata M., Neusser M., Kretzler M., Cohen C., Schlondorff D. (2008). Proteinuria and hyperglycemia induce endoplasmic reticulum stress. J. Am. Soc. Nephrol..

[27] Zhu G., Lee A. (2015). Role of the unfolded protein response, GRP78 and GRP94 in organ homeostasis. J. Cell Physiol..

[28] Kimura K., Jin J., Ogawa M., Aoe T. (2008). Dysfunction of the ER chaperone BiP accelerates the renal tubular injury. Biochem. Biophys. Res. Commun..

[29] Shore G., Papa F., Oakes S. (2011). Signalling cell death from the endoplasmic reticulum stress response. Curr. Opin. Cell Biol..

[30] Bijian K., Cybulsky A. (2005). Stress proteins in glomerular epithelial cell injury. Contrib. Nephrol..

[31] Sreedharan R., Van Why S. (2016). Heat shock proteins in the kidney. Pediatr. Nephrol..

[32] Lanneu D., Brunet M., Frisan E., Solary E., Fontenay M., Garrido C. (2008). Heat shock proteins: Essential proteins for apoptosis regulation. J. Cell. Mol. Med..

[33] Smoyer W., Gupta A., Mundel P., Ballew J., Welsh M. (1996). Altered expression of glomerular heat shock protein 27 in experimental nephrotic syndrome. J. Clin. Investig..

[34] Smoyer W., Ransom R. (2002). Hsp27 regulates podocyte cytoskeletal changes in an in vitro model of podocyte process retraction. FASEB J..

[35] Wadhwa P., Taira K., Kaul S. (2002). An Hsp70 family chaperone, mortalin/mthsp70/PBP74/Grp75: What, when, where?. Cell Stress Chaperones.

[36] Imasawa T., Rossignol R. (2013). Podocyte energy metabolism and glomerular diseases. Int. J. Biochem. Cell Biol..

[37] Che R., Yuan Y., Huang Y., Zhang A. (2014). Mitochondrial dysfunction in the pathophysiology of renal diseases. Am. J. Physiol. Ren. Physiol..

[38] Granquist A., Nilsson U., Ebefors K., Haraldsson B., Nystrom J. (2010). Impaired glomerular and tubular antioxidative defense mechanisms in nephrotic syndrome. Am. J. Physiol. Ren. Physiol..

[39] Tian N., Arany I., Waxman D., Baliga R. (2010). Cytochrome P450 2B1 gene silencing attenuates puromycin aminonucleoside-induced cytotoxicity in glomerular epithelial cells. Kidney Int..

[40] Gaull G. (1986). Taurine as a conditionally essential nutrient in man. J. Am. Coll. Nutr..

[41] Yamori Y., Taguchi T., Hamada A., Kunimasia K., Mori H., Mori M. (2010). Taurine in health and diseases: Consistent evidence from experimental and epidemiological studies. J. Biomed. Sci..

[42] Jong C., Ito T., Prentice H., Wu J., Schaffer S. (2017). Role of mitochondria and endoplasmic reticulum in taurine-deficiency-mediated apoptosis. Nutrients.

[43] Schaffer S., Kim H.W. (2018). Effects and mechanisms of taurine as a therapeutic agent. Biomol. Ther..

[44] Han H., Chesney R. (2012). The role of taurine in renal disorders. Amino Acids.

[45] Ito T., Schaffer S., Azuma J. (2012). The potential usefulness of taurine on diabetes mellitus and its complications. Amino Acids.

[46] Sarkar P., Basak P., Gosh S., Kundu M., Sil P. (2017). Prophylactic role of taurine and its derivatives against diabetes mellitus and its related complications. Food Chem. Toxicol..

[47] Chen W., Guo J., Zhang Y., Zhang J. (2017). The beneficial effects of taurine in preventing metabolic syndrome. Food Funct..

[48] Venkatesan N., Rao P., Arumugam V. (1993). Inhibitory effect of taurine on puromycin aminonucleoside-induced hyperlipidemia in rats. J. Clin. Biochem. Nutr..

[49] Feng Y., Li J., Yang Y., Yang Q., Lv Q., Gao Y., Hu J. (2013). Synergistic effects of taurine and l-arginine on attenuating insulin resistance hypertension. Adv. Exp. Med. Biol..

[50] Kerlin B., Waller A., Sharma R., Chanley M., Nieman M., Smoyer W. (2015). Disease severity correlates with thrombotic capacity in experimental nephrotic syndrome. J. Am. Soc. Nephrol..

[51] Moloney M.A., Casey R.G., O’Donnell D.H., Fitzgerald P., Thompson C., Bouchier-Hayes D.J. (2010). Two weeks taurine supplementation reverses endothelial dysfunction in young male type 1 diabetics. Diabetes Vasc. Dis. Res..

[52] Stacchiotti A., Lavazza A., Rezzani R., Borsani E., Rodella L., Bianchi R. (2004). Mercuric-chloride induced alterations in stress proteins distribution in rat kidney. Histol. Histopathol..

[53] Sitrin J., Suto E., Wuster A., Eastham-Anderson J., Kim J., Austin C., Lee W., Beherens T. (2017). The Ox40/Ox40 ligand pathway promotes pathogenic Th cell responses, plasmablast accumulation, and lupus nephritis in NZB/W F1 mice. J. Immunol..

[54] Stacchiotti A., Ricci F., Rezzani R., Li Volti G., Borsani E., Lavazza A., Bianchi R., Rodella L.F. (2006). Tubular stress proteins and nitric oxide synthase expression in rat kidney exposed to mercuric chloride and melatonin. J. Histochem. Cytochem..

[55] He C., Imai M., Song H., Quigg R., Tomlinson S. (2005). Complement inhibitors targeted to the proximal tubule prevent injury in experimental nephrotic syndrome and demonstrate a key role for C5b-9. J. Immunol..

[56] Stacchiotti A., Favero G., Giugno L., Lavazza A., Reiter R., Rodella L.F., Rezzani R. (2014). Mitochondrial and metabolic dysfunction in renal convoluted tubules of obese mice: Protective role of melatonin. PLoS ONE.

[57] Stacchiotti A., Favero G., Lavazza A., Golic I., Aleksic M., Korac A., Rodella L.F., Rezzani R. (2016). Hepatic macrosteatosis is partially converted to microsteatosis by melatonin supplementation in ob/ob mice nonalcoholic fatty liver disease. PLoS ONE.

[58] Patrakka J., Lahdenkari A., Koskimies O., Holmberg C., Wartiovaara J., Jalanko H. (2002). The number of podocyte-slit diaphragms is decreased in minimal change nephrotic syndrome. Pediatr. Res..

[59] Sanz A., Santamaria B., Ruiz-Ortega J., Egido J., Ortiz A. (2008). Mechanisms of renal apoptosis in health and disease 2. J. Am. Soc. Nephrol..

[60] Patergnani S., Suski J., Agnoletto C., Bononi A., Bonora M., De Marchi E., Giorgi C., Marchi S., Missiroli S., Poletti F. (2011). Calcium signaling around mitochondria associated membranes (MAMs). Cell Commun. Signal..

[61] De Zeeuw D., Remuzzi G., Parving H., Keane W., Zhang Z., Shahinfar S., Snapinn S., Cooper M., Mitch W., Brenner B. (2004). Proteinuria, a target for renoprotection in patients with type 2 diabetic nephropathy: Lessons from RENAAL. Kidney Int..

[62] Abbate M., Zoja C., Remuzzi G. (2006). How does proteinuria cause progressive renal damage?. J. Am. Soc. Nephrol..

[63] Cybulsky A. (2010). Endoplasmic reticulum stress in proteinuric kidney disease. Kidney Int..

[64] Takeuchi S., Hiromura K., Tomioka M., Takahashi S., Sakairi T., Maeshima A., Kaneko Y., Kuroiwa T., Nojima Y. (2010). The immunosuppressive drug mizoribine directly prevents podocyte injury in puromycinaminonucleoside nephrosis. Nephron Exp. Nephrol..

[65] Hammadi M., Oulidi A., Gackiere F., Katsogiannou M., Slomianny C., Roudbaraki M., Dewailly E., Delcourt P., Lepage G., Lotteau S. (2013). Modulation of ER stress and apoptosis by endoplasmic reticulum calcium leak via translocon during unfolded protein response: Involvement of GRP78. FASEB J..

[66] Rovetta F., Stacchiotti A., Consiglio A., Cadei M., Grigolato P., Lavazza A., Rezzani R., Aleo M.F. (2012). ER signaling regulation drives the switch between autophagy and apoptosis in NRK-52E cells exposed to cisplatin. Exp. Cell Res..

[67] Zhang Y., Ren Y., Liu Y., Gao K., Liu Z., Zhang Z. (2017). Inhibition of starvation-triggered endoplasmic reticulum stress, autophagy, and apoptosis in ARPE-19 cells by taurine through modulating the expression of calpain-1 and calpain-2. Int. J. Mol. Sci..

[68] Wen Y., Zhan Y., Liu H., Zhao T., Yang L., Zhang H., Dong X., Li P. (2015). Yi Qi Qing Re Gao formula ameliorates puromycin aminonucleoside-induced nephrosis by suppressing inflammation and apoptosis. BMC Complement. Altern. Med..

[69] Mallipattu S., He H. (2015). The beneficial role of retinoids in glomerular disease. Front. Med..

[70] Prentice H., Modi J., Wu J. (2015). Mechanisms of neuronal protection against excitotoxicity, endoplasmic reticulum stress, and mitochondrial dysfunction in stroke and neurodegenerative diseases. Oxid. Med. Cell. Longev..

[71] Cocchiaro P., de Pasquale V., Della Morte D., Taffuri S., Avallone L., Pizard A., Moles A., Pavone L. (2017). The multifaceted role of the lysosomal protease cathepsins in kidney disease. Front. Cell Dev. Biol..

[72] Mozaffari M.S., Abdelsayed R., Patel C., Wimborne H., Liu J.Y., Schaffer S.W. (2010). Differential effects of taurine treatment and taurine deficiency on the outcome of renal ischemia reperfusion injury. J. Biomed. Sci..

[73] Shimada K., Jong C., Takahashi K., Schaffer S. (2015). Role of ROS production and turnover in the antioxidant activity of taurine. Adv. Exp. Med. Biol..

[74] Chebotareva N., Bobkova I., Shilou E. (2017). Heat shock proteins and kidney disease: Perspectives of HSP therapy. Cell Stress Chaperones.

[75] Bravo R., Vicencio J., Parra V., Troncoso R., Munoz J., Bui M., Quiroga C., Rodriguez A., Verdejo H., Ferreira J. (2011). Increased ER-mitochondrial coupling promotes mitochondrial respiration and bioenergetics during early phases of ER stress. J. Cell Sci..

[76] El Idrissi A. (2008). Taurine increases mitochondrial buffering of calcium: Role of neuroprotection. Amino Acids.

[77] Fang L., Xie D., Xian W., Cao H., Su W., Yang J. (2013). Involvement of endoplasmic reticulum stress in albuminuria induced inflammasome activation in renal proximal tubular cells. PLoS ONE.

[78] Zhuang Y., Ding G., Zhao M., Bai M., Yang L., Ni J., Wang R., Jia Z., Huang S., Zhang A. (2014). NLRP3 inflammasome mediates albumin-induced renal tubular injury through impaired mitochondrial function. J. Biol. Chem..

[79] Qureshi M., Haynes C., Pellegrino M. (2017). The mitochondrial unfolded protein response: Signaling from the powerhouse. J. Biol. Chem..

