# Supplementary figures and images for: Taurine Supplementation Alleviates Puromycin Aminonucleoside Damage by Modulating Endoplasmic Reticulum Stress and Mitochondrial-Related Apoptosis in Rat Kidney
Source: Nutrients. 2018 May 29;10(6):689. doi: 10.3390/nu10060689 (PMC6024760; doi:10.3390/nu10060689)

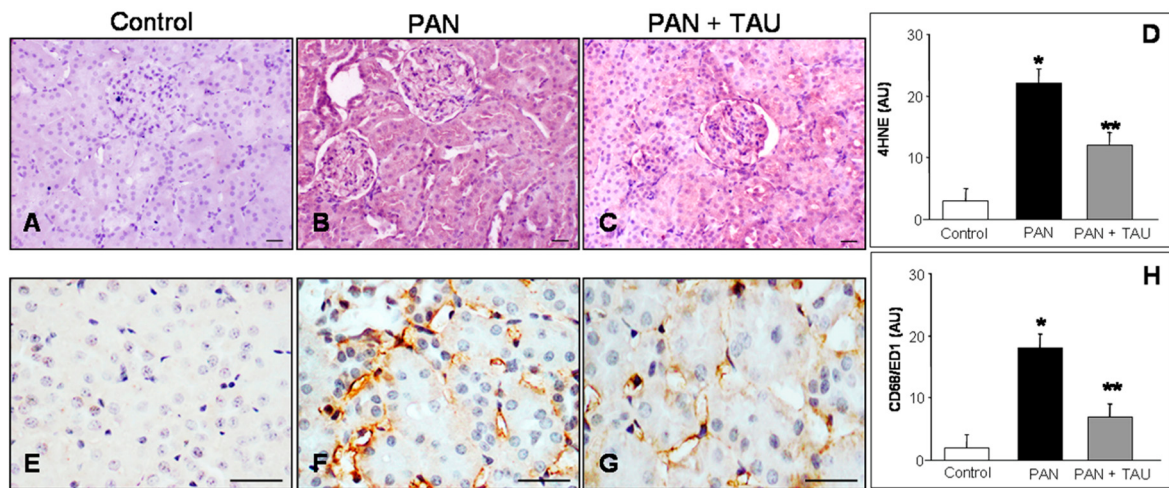

Figure S1: 4HNE and CD68/ED1 renal expression.

Supplement: Supplementary file 1 [file nutrients-10-00689-s001.pdf]
